# Climate mediates color morph turnover in a species exhibiting alternative reproductive strategies

**DOI:** 10.1038/s41598-022-12300-7

**Published:** 2022-05-19

**Authors:** Matthew S. Lattanzio

**Affiliations:** Department of Organismal and Environmental Biology, Christopher Newport University, Newport News, VA 23606 USA

**Keywords:** Sexual selection, Behavioural ecology, Climate-change ecology, Ecology, Ecophysiology

## Abstract

Sexual selection is considered the primary driver of morph turnover in many color polymorphic taxa, yet the potential for other factors (like climate) to contribute to polymorphism maintenance and evolution remains unclear. Appreciation for a role of environmental conditions in the maintenance and evolution of color polymorphisms has grown in recent years, generating evidence suggesting that color morphs linked to sexual selection may also diverge in climate sensitivity. Focusing on the three color components contributing to the male tree lizard (*Urosaurus ornatus*) color morphs, I reveal a marked concordance between patterns of turnover over space and time, with a general affinity of orange- and yellow-colored males to hotter, more variable conditions, and blue colored males to wetter, cooler conditions. An assessment of long-term turnover in the blue color component in response to recent climate change over the past 60 years reinforces these findings. Overall, behavioral asymmetries attributed to sexual selection likely expose competing morphs to divergent environmental conditions in heterogeneous habitats, creating opportunity for natural selection to shape climate sensitivities that also drive turnover in morph color composition. Ultimately, these processes may favor stark asymmetries in morph persistence over the coming decades.

## Introduction

Color polymorphisms offer fascinating and compelling models for testing drivers of intraspecific diversification and ecological speciation^1,2^, particularly those linked with alternative reproductive strategies (ARS)^1^. Across a diverse array of vertebrates and invertebrates alike, ARS color polymorphisms are associated with discrete variation in resource holding potential and reproductive quality among competing male color morphs^3–7^. Physiological differences among morphs in social dominance and behavior are then expected to shape differential access to and preference by potential female mates^8,9^. As a result, treatment of potential factors shaping the evolution and maintenance of ARS color polymorphisms is usually constrained to fit within a sexual selection framework.

This emphasis on sexual selection has hindered our appreciation of the potential breadth of other factors that may influence the maintenance and evolution of ARS color polymorphic systems (likewise, ecological polymorphisms may also interact with sexual selection processes; see^10^). Natural selection should also influence color morph maintenance and evolution in taxa exhibiting ARS because morph differences in underlying hormone function and levels influence their ability to perform ecologically relevant physiological functions like bite force^11^ and locomotor performance^12,13^. Morph differences in social behavior also influence how they interact with available environmental resources, including their foraging strategy^14–16^ and microhabitat use^17^, as well as how morphs cope with shifts in resource availability^18,19^. For example, aggressive, dominant morphs in the tree lizard (*Urosaurus ornatus*) and their congener (*U. graciosus*) consistently exploit preferred microhabitat types despite shifts in their availability whereas other morphs exhibit more generalist and/or plastic ecological roles^6,15,20^. Despotic spatial dispersion among color morphs with respect to preferred resources in the wild has also been observed, whereby dominant morphs monopolize access to high-quality microhabitats relative to other males^20,21^.

Appreciation for a role of environmental conditions in the maintenance and evolution of ARS color polymorphisms has also grown over the past several years, fostering an even deeper understanding of how evolution operates in these systems^17,22–24^. As a result, there is mounting evidence to suggest that morphs may also diverge in climatic sensitivity^17,22,23,25^, leading to morph-specific differences in survival across an environmental gradient. Identifying such divergence in ARS systems is crucial because it may help explain peculiarities that arise when attempting to reconcile observations of morph-occupancy patterns (e.g., mono- and di-morphic populations, and/or clinal variation in morph frequencies) made across a species’ range or within a single population over time that violate predictions of the sexual selection model to which they are expected to adhere^23,24,26^.

Prior assessments of climate-mediated morph turnover fall into one of two categories: spatial efforts that capitalize on available bioclimatic variables^17,24,25^, or approaches that rely on climate data interpolated to the timing of the study and/or life history of the focal species^23,27^. Because bioclimatic variables represent 30-year averages of temperature and precipitation conditions spanning 1970–2000^28^, inferences on their impacts on short-lived taxa (e.g., polymorphic lizards) should be drawn with caution. Specifically, although bioclimatic variables are likely useful for long-lived species (e.g.,^29^), many generations (and morph cycles) have likely passed during that 30-year period, and several more since those climate data were last measured. These considerations reveal a crucial temporal disparity between fluctuations in morph frequencies and their presumed climatic driver(s) in studies relying on bioclimatic data.

Both temperature and precipitation data that match the timing of the study itself and the focal species’ life history are therefore essential if we aspire to draw meaningful conclusions about morph-climate interactions. To my knowledge, there have been only two studies on color polymorphic species that addressed this consideration^23,27^. In a study on a non-ARS color polymorphic species, spatial shifts in dark and light morph sparrowhawk (*Accipter melanoleucus*) morph proportions across the species’ range in South Africa coincided with shifts in breeding season climate (temperature and precipitation)^27^. However, because morph cycling is a fundamentally temporal process in nature, time-series data are also needed to fully ascertain the extent that morph turnover patterns relate to concomitant climate shifts (if at all). Yet, although time-series climate data were incorporated into a study on side-blotched lizards (*Uta stansburiana*, a species exhibiting an ARS color polymorphism^26^), treatment of climate was limited to an indirect measure of temperature, the hours of activity restriction^23^. Thus, the direct impact of temperature on color morph turnover, as well as any impact of other temperature or precipitation descriptors, remain unclear. Ideally, the assessment of climate-mediated turnover in any color polymorphic species (ARS or otherwise) should involve consideration of a spatial climatic gradient (to capture ecogeographic trends) as well as both short-term (to capture annual morph frequency shifts) and long-term (to capture broader evolutionary trends) temporal climatic gradients. For ARS color polymorphic species in particular, concordance between climate association patterns over space and time would provide robust evidence in favor of a novel role for climate to also influence morph turnover.

**Figure 1:**
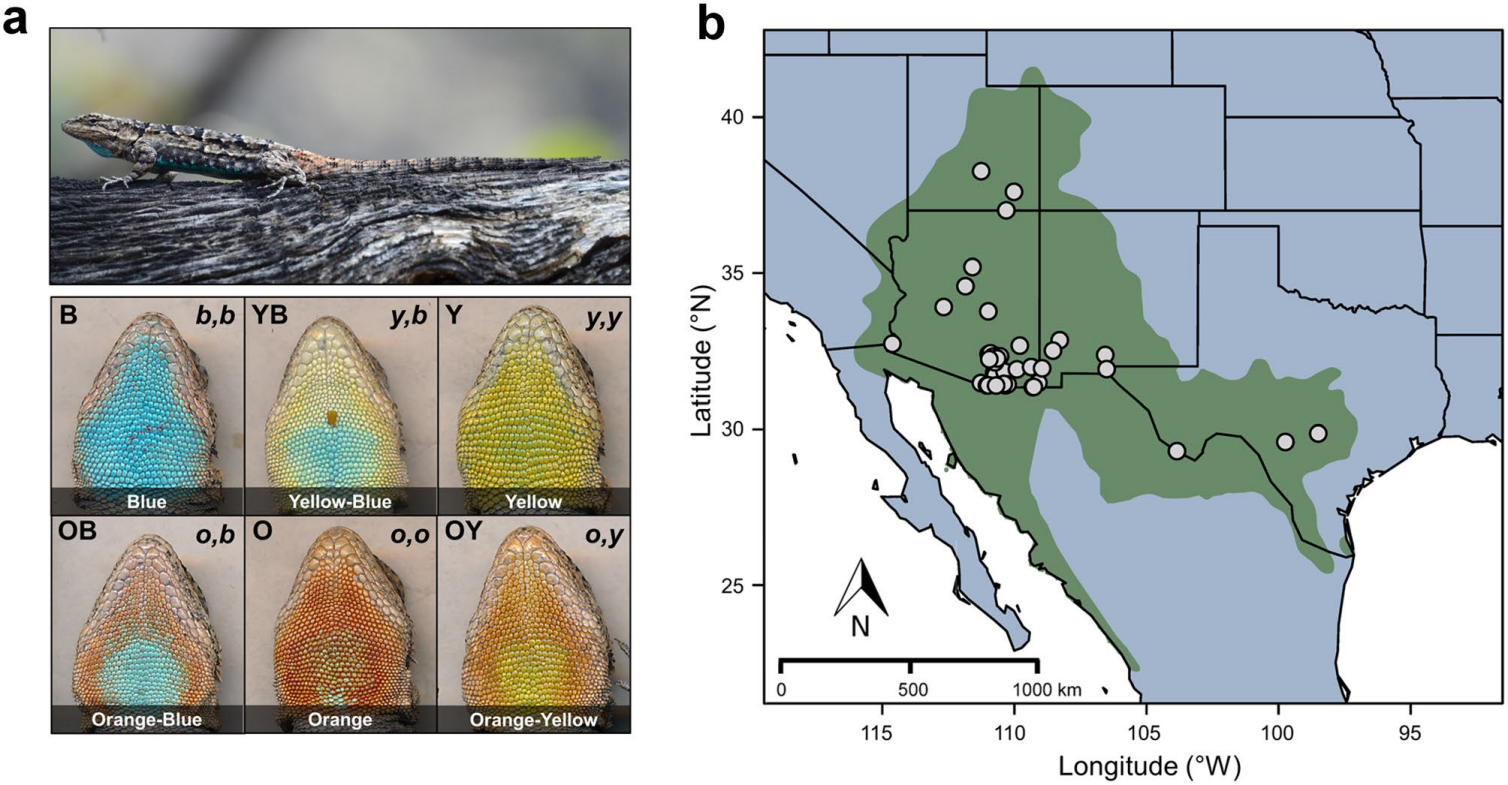
(panel **a**) Representative image of adult male *U. ornatus* and scans of the six color morphs. Overlays indicate morph coding consistent with phenotypic (uppercase lettering, indicative of observed discrete badge coloration) and genotypic (lowercase italicized lettering, indicative of putative alleles underlying badge color) approaches to characterizing morph coloration^22,26,49^. Note that in some populations, the central spot color of yellow-blue and orange-blue morphs may appear green [e.g.,^46^], depending on the amount of yellow or orange pigmentation over the structural blue color underneath. (panel **b**) Map of the 58 localities surveyed for *U. ornatus* for the spatial dataset. The green shaded region represents the species’ full geographic distribution in North America.

In this study I capitalize on a long-term dataset on the color polymorphic tree lizard (*U. ornatus*, Fig. 1a) to test the hypothesis that color polymorphisms linked to alternative mating strategies also exhibit divergence in climate sensitivity. Focusing on the three color components of the polymorphism in this species, I first evaluated spatial turnover in male color components among 58 localities distributed through a climatically variable portion of the species’ geographic range (Fig. 1b). I then assessed the concordance of those findings with temporal patterns of climate-mediated turnover in color components across three localities surveyed over a seven-year period. Finally, I also considered patterns of long-term turnover in color composition in relation to recent climate change over the past 60 years. Overall, my findings provide strong support for divergence in sensitivity to climate conditions linked to throat coloration that should also contribute to the maintenance and evolution of a color polymorphism. As the impacts of climate change accelerate, these patterns may also generate asymmetries in morph persistence over the coming decades.

## Results

### Spatial turnover

**Figure 2:**
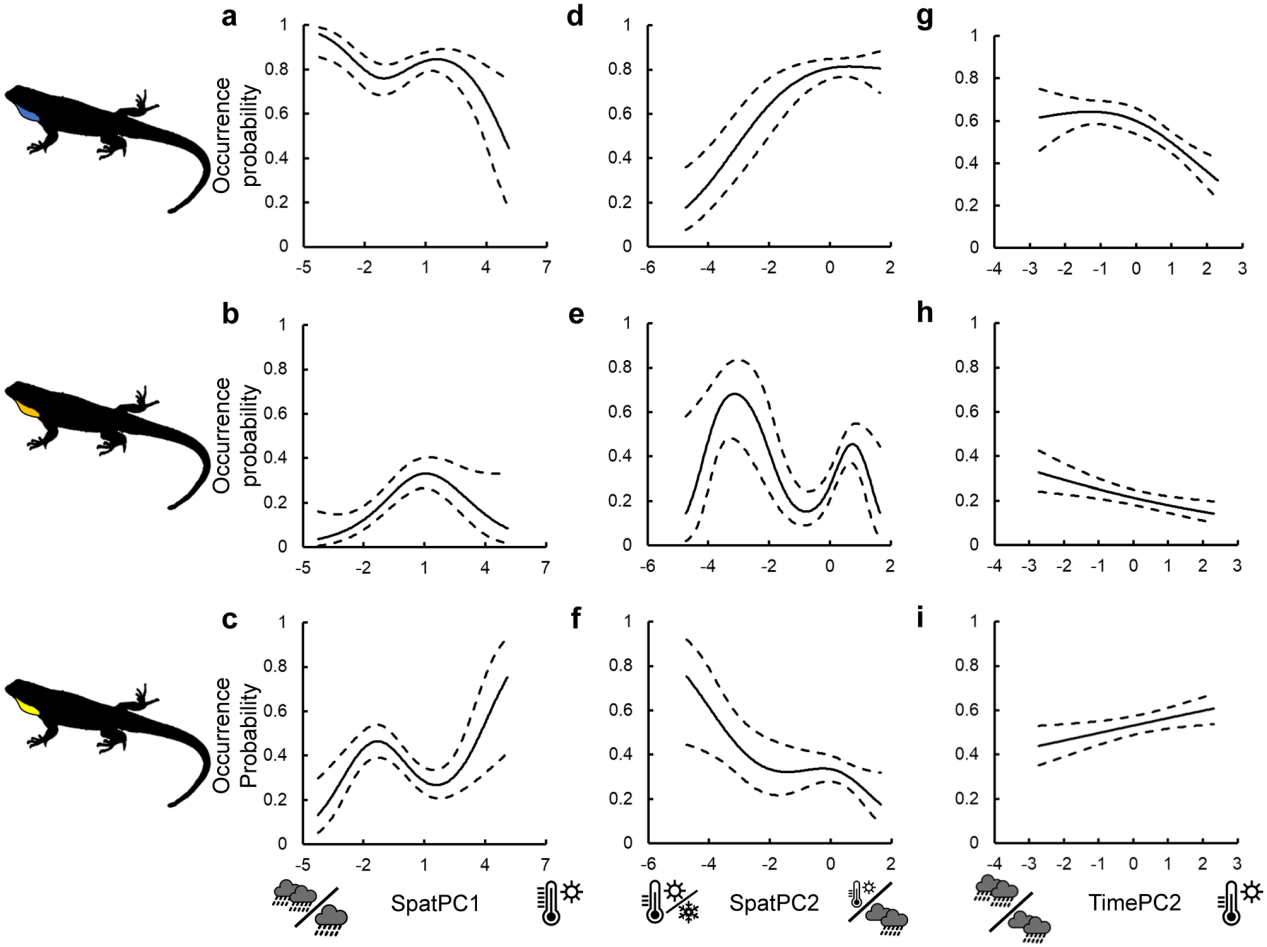
Predicted associations between male *U. ornatus* color components and climate PCs (Spatial dataset: blue [panels **a**, **d**], orange [panels **b**, **e**], and yellow [panels **c**, **f**]; Time-series dataset: blue [panel **g**], orange [panel **h**], yellow [panel **i**]) retained in the best fit model for each phenotype (broken lines: 95% CI). Model *R*^2^ = 0.719, 0.598, and 0.406 for the spatial dataset, and *R*^2^ = 0.697, 0.405, and 0.25 for the time-series dataset.

Blue morphs occurred at over twice the frequency of any other morph overall (χ2 = 413.1, df = 5, *p* < 0.001). Climate conditions varied extensively across the 58 localities (Table 1); with average temperatures ranging 18.33–32.37 °C and active season precipitation ranging 37.55–536.01 mm (Supplementary Table S1). Patterns of color component turnover across the studied range reveal a marked concordance to climatic gradients (Fig. 2a-f). Of all possible models, the best-fit model explaining the distribution of blue color components retained both SpatPC1 and SpatPC2 as predictors (GAM, AICc = 212.6, *R*^2^ = 0.719; see Table 2). Blue throat coloration was most strongly associated with exploitation of wetter and cooler habitats (SpatPC1), as well as warmer habitats with more-variable rainfall throughout the year (SpatPC2) (Fig. 2a, d). Occupancy by the orange color component was also best explained by a model including SpatPC1 and SpatPC2 terms (GAM, AICc = 246.5, *R*^2^ = 0.598; see Table 2). Unlike blue coloration, orange coloration was associated with a more generalist climate strategy: peak occurrence occurred in somewhat warmer and drier habitats for SpatPC1, and for SpatPC2, there was a peak representing cooler, seasonal temperature habitats experiencing less variable precipitation, and a secondary peak in warmer environments with more variable precipitation rates (Fig. 2b, e). The best-fit model explaining variation in the spatial distribution of the yellow color component also included SpatPC1 and SpatPC2 (GAM, AICc = 240.4, *R*^2^ = 0.406; see Table 2). Lizards with yellow coloration were most common in relatively drier and hotter habitats, or cooler habitats with more variable temperatures but constant precipitation rates overall (Fig. 2c, f). Complementary GAM analyses applied to only those localities with ≥ 10 males (*n* = 28) to account for potential bias introduced by low sample sizes produced qualitatively similar results to those described above (Supplementary Table S6).

**Table 1:** Loadings of climate variables on the first two component axes of separate principal components analyses (PCAs) applied to the spatial and time-series datasets, respectively.

Climate variable—Spatial dataset	Icon label	PC1	PC2
Mean daytime air temperature (°C)		0.389	**0.447**
Maximum daily air temperature (°C)		**0.474**	0.231
Standard deviation of mean daytime air temperature (°C)		0.258	− **0.661**
Mean daily precipitation rate (mm/day)		**− 0.507**	0.18
Total precipitation during the active season (mm)		**− 0.505**	0.241
Coefficient of variation of mean daily precipitation rate (%)		0.214	**0.468**
Eigenvalue		1.83	1.28
Variance explained (%)		55.6	27.4
Cumulative variance (%)		55.6	83

Climate variable—Time-series dataset	Icon label	PC1	PC2
Mean daytime air temperature (°C)		**0.422**	**0.437**
Maximum daily air temperature (°C)		**0.492**	0.189
Standard deviation of mean daytime air temperature (°C)		0.388	**− 0.409**
Mean daily precipitation rate (mm/day)		**0.461**	− 0.291
Total precipitation during the active season (mm)		0.252	**− 0.589**
Coefficient of variation of mean daily precipitation rate (%)		− 0.391	**− 0.418**
Eigenvalue		1.63	1.51
Variance explained (%)		44.2	38
Cumulative variance (%)		44.2	82.2

Significant values are in [bold].

Each PCA was performed on the correlation matrix of raw climate data (see "Methods" for variable details). Strong loadings ( >|0.402| for the Spatial dataset and >|0.404| for the Time-series dataset) are presented in bold typeface.

**Table 2:** Effects of climate on the occurrence of blue, orange, and yellow color components on the throat badge of male lizards in the spatial dataset.

Model	Syntax	AICc	ΔAICc	*w*_*i*_(AICc)	*R*^2^_adj_	*χ*^2^	P
*Blue color component*							
BL12	~ s(SpatPC1) + s(SpatPC2)	212.6	0	0.994	0.719	72.28	< 0.001
BL2	~ s(SpatPC2)	222.7	10.15	0.006		56.91	< 0.001
BL1	~ s(SpatPC1)	258.1	45.51	< 0.001		21.99	< 0.001
BL_NULL_	~ 1	273.6	61.03	< 0.001		–	–
*Orange color component*							
OR12	~ s(SpatPC1) + s(SpatPC2)	246.5	0	0.848	0.598	52.2	< 0.001
OR2	~ s(SpatPC1)	249.9	3.45	0.152		45.53	< 0.001
OR1	~ s(SpatPC2)	282.3	35.83	< 0.001		11.03	< 0.001
OR_NULL_	~ 1	289.7	43.28	< 0.001		–	–
*Yellow color component*							
YE12	~ s(SpatPC1) + s(SpatPC2)	240.4	0	0.769	0.406	29.76	< 0.001
YE2	~ s(SpatPC2)	243.8	3.39	0.141		20.12	< 0.001
YE1	~ s(SpatPC1)	244.7	4.31	0.089		19.25	< 0.001
YE_NULL_	~ 1	257.4	17.08	< 0.001		–	–

A series of candidate generalized additive models were used to evaluate the effect of climate (SpatPC1 and SpatPC2) on color component frequency. *Nagelkerke’s R*^2^ values (adjusted for low sample sizes) are provided for the best-fit model for each color component (i.e., lowest AICc and highest *w*_*i*_[AICc]). Likelihood-ratio tests compare model fit to a null model. See "Methods" for variable descriptions, and Fig. 1b for a map of study localities.

### Temporal turnover

Blue morphs were almost twice as common as any other morph in the time-series dataset (χ2 = 252.5, df = 5, *p* < 0.001). Climate conditions were less variable in this dataset (see Table 1) and overlapped with moderate values observed in the spatial dataset, with average temperatures ranging 23.11–24.79 °C and active season precipitation ranging 283.5–325.9 mm (Supplementary Table S2). Some patterns of color component turnover over time mirrored those identified in the spatial dataset (Fig. 2g-i). For the blue color component, both the full model and the model retaining only TimePC2 were within 2 AICc units of one another; I therefore interpreted the simpler model as best fit (GAM, AICc = 104.7, *R*^2^ = 0.697; see Table 3). There was a greater probability of encountering males with blue coloration during cooler, wetter years with more seasonal conditions (Fig. 2g). For the orange color component, the best-fit model only retained TimePC2 as a predictor (GAM, AICc = 83.1, *R*^2^ = 0.405; see Table 3), and suggested a high occupancy probability of encountering males with orange coloration during cooler and wetter years (Fig. 2h). Finally, of all candidate models, the best-fit model for the yellow color component only retained TimePC2 (AICc = 103.4, *R*^2^ = 0.25; see Table 3), with occupancy probability highest in warmer, drier years (Fig. 2i).

**Table 3:** Effects of climate on the occurrence of blue, orange, and yellow color components on the throat badge of male lizards in the time-series dataset.

Model	Syntax	AICc	ΔAICc	*w*_*i*_(AICc)	*R*^2^_adj_	*χ*^2^	P
*Blue color component*							
BL2	~ s(TimePC2)	104.7	0	0.512	0.697	24.96	< 0.001
BL12	~ s(Time PC1) + s(TimePC2)	104.8	0.09	0.488		29.42	< 0.001
BL1	~ s(TimePC1)	123.2	18.49	< 0.001		8.23	0.016
BL_NULL_	~ 1	126.6	21.86	< 0.001		–	–
*Orange color component*							
OR2	~ s(TimePC2)	83.1	0	0.678	0.405	10.68	0.005
OR12	~ s(TimePC1) + s(TimePC2)	85.7	2.63	0.183		10.51	0.015
OR1	~ s(TimePC1)	86.9	3.78	0.103		4.95	0.026
OR_NULL_	~ 1	88.9	5.83	0.037		–	–
*Yellow color component*							
YE2	~ s(TimePC2)	103.4	0	0.656	0.25	5.98	0.014
YE12	~ s(TimePC1) + s(TimePC2)	106	2.66	0.174		6.07	0.048
YE_NULL_	~ 1	106.9	3.53	0.113		–	–
YE1	~ s(TimePC1)	108.3	4.87	0.057		6.28	0.1

A series of candidate generalized additive models were used to evaluate the effect of climate (TimePC1 and TimePC2) on color component frequency. Nagelkerke’s *R*^2^ values (adjusted for low sample sizes) are provided for the best-fit model for each allele (i.e., lowest AICc and highest *w*_*i*_[AICc]). Likelihood-ratio tests compare model fit to a null model. See "Methods" for variable descriptions.

### Climate change: long-term patterns

**Figure 3:**
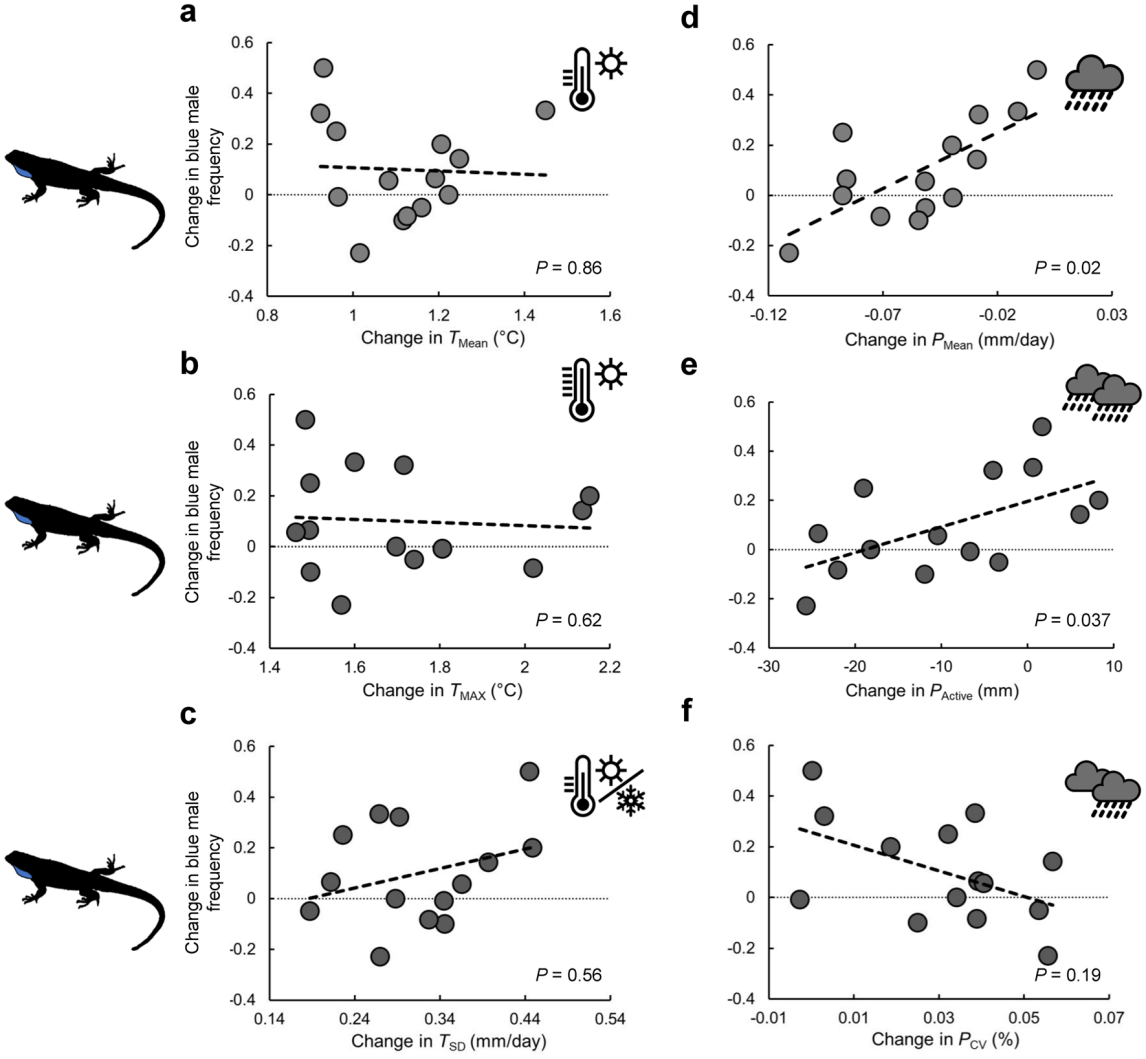
Associations between long-term shift in composition (proportion of males exhibiting blue coloration) of 14 populations of *U. ornatus* from the spatial dataset and shifts in temperature (panels **a**-**c**) and precipitation (panels **d**-**f**) due to recent climate change (see "Methods" for variable descriptions). Trendlines are for illustrative purposes only, and *p*-values are results of correlation tests (see "Results" Section).

Over the past ~ 60 years, daily temperatures rose between 0.92 and 1.45 °C across the 14 localities, whereas total precipitation during the active season decreased at all but four localities (range: − 25.7–8.23 mm) (Fig. 3). Proportions of males with blue coloration varied similarly across the 14 localities between both historical and present-day surveys (mean [range]: past, 0.72 [0.43,1]; present-day, 0.82 [0.5,1]). However, the population composition of each locality exhibited a marked shift over time that coincided in part with recent climate change (*T*_Mean_: *r* = − 0.055, *p* = 0.86; *T*_SD_: *r* = 0.169, *p* = 0.56; *T*_Max_: *r* = − 0.147, *p* = 0.62; *P*_Mean_: *r* = 0.622, *p* = 0.02; *P*_CV_: *r* = − 0.375, *p* = 0.18; *P*_Active_: *r* = 0.569, *p* = 0.037). In general, temporal fluctuations in population composition of blue colored males coincided with long-term shifts in daily and active season precipitation (Fig. 3d-e).

## Discussion

For ARS color polymorphisms, morph turnover is attributed to negative frequency-dependent sexual selection that ensures persistence of all morphs within a population over time^26^. However, recent studies support that morph behavioral asymmetries interact with environmental heterogeneity, generating divergence among competing morphs in ecological traits relevant for natural selection^15,17^. The spatial dispersion of color morphs appears to also have a climatic component^23^^,^^24^^,^^27^, this study. Here I provide robust evidence for climate-mediated patterns of turnover in morph coloration that were generally consistent over space and time: the association of blue throat coloration with exploitation of wetter and cooler conditions, the association of orange coloration with exploitation of more seasonal conditions, and the association of yellow coloration with exploitation of drier, hotter conditions. The broad range of climate conditions in my spatial dataset also revealed additional contrasts between the phenotypes: as long as precipitation was variable, orange males could exploit cooler conditions and blue males could exploit warmer conditions. In contrast, males that had yellow coloration were more tolerable to variation in temperature than precipitation. For blue males, these processes appear to have also driven evolutionary shifts in population composition over the past several decades in response to recent climate change. Overall, these findings support my hypothesis, revealing that climate variability also mediates color polymorphism turnover in a species exhibiting alternative mating strategies.

Given its consistent association with cooler and wetter conditions over space and time, blue coloration may be linked to a reduced tolerance to desiccation. Hillman and Gorman^30^ showed marked variation in desiccation tolerance (measured as maximum survival time) among several lizard species, and a general pattern for species exposed to wetter and/or more-homogenous climate climates to be less-tolerant to desiccation than other lizards^30^. This association may be a general pattern among squamates^31^, and salamanders as well^32^. Moreover, the high degree of variability in water loss rates among closely related species in^30^ supports that these rates are evolutionarily labile. Thus, it may be possible that color morphs within a wide-ranging species like *U. ornatus* that experiences broad climatic gradients may also diverge in desiccation tolerance (or other climatic sensitivity). Intraspecific variation in desiccation tolerance is not a novel concept, even for lizards. For example, in the mountain spiny lizard (*Sceloporus jarrovii*), juvenile males exhibit lower desiccation tolerance (measured as evaporative water loss rate) compared to females^33^, which, along with their faster growth rates^34^, should constrain their daily activity patterns in the short-term and make them more susceptible to climate warming in the long-term. In my study, the associations between blue colored males and precipitation were more consistent than those with temperature across all three datasets, with peak occupancy occurring in wetter conditions over both space and time. A key hypothesis drawing from these observations would be that blue colored males exhibit lower desiccation tolerance than other morphs. Currently, little is known about desiccation tolerance in *U. ornatus* aside from it being lower than several *Anolis* species^30^. Assessing morph-specific divergence in desiccation tolerance as well as thermal traits would thus be a fruitful area for further inquiry in *U. ornatus* as well as others where morph occupancy appears environment-dependent^17,24^.

Clear environmental clines in morph occurrence patterns along environmental gradients that appear driven by consistent morph-climate associations, and/or variation in the strength of sexual selection, have been demonstrated in prior studies^25,27,35^. However, such patterns in *U. ornatus* color component occurrence across both latitude and elevation (two common biogeographic proxies for environmental clines) are lacking or inconsistent (Supplementary Fig. S4). For example, I observed blue males at all but one locality in my study (Natural Bridges National Monument, UT), which may be due to the high variability in temperature at that locality (sd = 9.3 °C) relative to others (overall sd range: 6.5–9.5 °C), which should pose a challenge for blue males (Fig. 2d). However, blue males nonetheless persisted in hotter, more arid macrohabitats (e.g., Yuma, AZ), and orange and yellow males persisted in cooler, wetter macrohabitats (e.g., Tonto National Forest, AZ). These observations support that the impacts of temperature and precipitation conditions on each color component (and morph) may depend on local ecological factors. To that end, microhabitat selection is one well-known ecological mechanism used by ectotherms to buffer climatic challenges^36,37^. Divergence in microhabitat usage across localities by the five common wall lizard (*Podarcis muralis*) color morphs across localities also exposes them to different microclimate conditions^17^ which coincide with the morph-climate associations documented by^24^. In *U. ornatus*, males that exhibit orange or yellow coloration are flexible in perch use unlike males exhibiting blue coloration, which exhibit a consistent preference for trees over snags (dead trees) or rocks (Supplementary Fig. S2). Relative to those other microhabitat types, trees are sources of higher relative humidity and provide a greater thermal buffer against extreme conditions via shade and heterogeneity in vertical structure. As a result, loss of living trees can lead to higher temperatures and decreased relative humidity^38^. In regions exploited by *U. ornatus*, canopy cover of living trees also reduces ground (soil) temperature and enhances moisture levels compared to surrounding open-canopied areas^39^. Thus, occupancy of habitats over gradient of climate variability by blue males may be contingent upon tree availability to buffer against hotter and drier conditions. The climate association patterns of yellow and orange-yellow morph *P. muralis* mirror my findings for blue males in terms of climate associations (based on bioclimatic variables, see^24,28^). The geographic constraints on orange and orange-yellow *P. muralis* morphs in^24^ were attributed to potential physiological constraints for orange morphs (which may also relate to desiccation tolerance^24^;) that may carry over to orange-yellow males as well. If the lack of geographic restriction by blue-colored *U. ornatus* (or any color component or morph; Supplementary Fig. S3) in my study is related to their microhabitat use differences (Supplementary Fig. S2), the extent that color morphs of other species (like *P. muralis*) diverge in within-locality microhabitat preferences is worthy of future investigation.

In contrast, orange and yellow males may thrive in more-extreme environments across the species’ range given their climatic affinities and flexible perch use (Fig. 2, Supplementary Fig. S2). For example, the frequency of yellow male *U. ornatus* can surpass blue male frequency in more open-canopied (and thus likely hotter and drier^39^,) areas compared to other habitats^20^. Orange-colored males appear to be flexible in microhabitat use^20^ and mating strategy, becoming more sedentary and less nomadic during more productive years compared to drought years^40^. This conditional behavior switch is assumed to be an adaptive physiological response to shifts to the availability of preferred microhabitats (trees) and/or prey^40^ which may enhance their ability to persist across varying degrees of habitat heterogeneity. The capacity of orange-colored males to tolerate variable (seasonal) temperature and precipitation conditions should benefit them in this regard (Fig. 2e). Moreover, there is also the potential for climate sensitivities of different color components to interact in bicolor morphs, generating unique ecogeographic outcomes. For example, despite geographic constraints on other orange phenotypes, *P. muralis* orange-white morphs are common throughout the species’ overall distribution^24^, suggesting white coloration may override orange in terms of phenotypic climate sensitivities. In my study, many localities were characterized by relatively high frequencies of bicolor *U. ornatus* morphs compared to blue morphs (see Fig. S3). For these males, having yellow and/or orange coloration in addition to blue coloration (i.e., yellow-blue and orange-blue morphs) may override any ecogeographic constraints associated with the blue color component (e.g., facilitating greater use of more open-canopy rock and snag microhabitats). Future investigations into how the relationship between climate sensitivity, physiology (e.g., desiccation tolerance), and ecology (e.g., perch use) of each color component manifests in bicolor *U. ornatus* morphs will be necessary to address this consideration.

The results of spatial morph environmental association studies^17,24^ support the prediction that temporal variation in climate conditions should also play an important role in color morph turnover. Indirect support for this prediction draws from a previous study on *U. stansburiana*, whereby the hours of restriction (an indirect proxy for temperature) was found to influence morph turnover over time^23^. My time-series data for *U. ornatus* provide additional support for this prediction, whereby the occurrence of males exhibiting blue and orange color components increased in frequency during cooler and wetter years (in general), and yellow-colored males becoming more common in hotter and drier years. My findings also reveal strikingly clear overlap in climate-turnover patterns over space and time (even when accounting for lower sample sizes in the spatial dataset, Supplementary Table S6). Cross-generational data on changes in the frequency of blue males to recent climate change further support that the patterns of spatial and temporal turnover by *U. ornatus* males are adaptive responses to environmental variability. Over the course of many generations (23–51 generations; based on a mean generation time of 1.2 years for *U. ornatus* in Arizona, see^41^), the frequency of blue-colored males increased in populations at localities that exhibited minimal reductions in mean daily precipitation rate or total active season precipitation as well as localities that experienced an increase in active season precipitation over time due to recent climate change (Fig. 3d-e). Given the increased frequency of droughts in western North America and predictions of future climatic shifts towards greater aridity^42^, blue-colored males should be at a disadvantage as climate change progresses relative to other male phenotypes.

Importantly, climate is clearly not the sole driver of morph turnover in color polymorphic species, and so other factors may confound that prediction (and contribute to variable morph population compositions throughout the species’ range; Supplementary Fig. S3). Complicating things further is the fact that unlike in male *U. stansburiana*, all known monomorphic populations of *U. ornatus* are blue and not orange (Supplementary Fig. S3; see also^43^), suggesting that monomorphic orange or yellow populations may be unable to persist in the long-term. Social interactions may drive this outcome given the higher frequency of fighting (as evidenced by bite marks on captured animals and staged male-male contests in a laboratory setting) observed in a locality with abundant yellow males^20^. Thus, instability in the morph social hierarchy at a locality may arise as the frequency of dominant blue males declines relative to yellow males. Although frequency-dependent cycling is expected under a purely sexual selection model^26^, my findings demonstrate morph turnover patterns over space and time may also be related to climate variability, revealing an underappreciated but crucial environmental axis along which color morphs linked to alternative mating strategies may also diversify (Fig. 2).

Outside of sexual selection, explanations for spatial or temporal divergence in morph dispersion may include the presence of complex, morph-specific climatic associations, a generalized environmental cline in population composition, and/or variation in population genetic structuring^24^. Despite the overlap of morph-climate associations between datasets, a clear and consistent environmental cline in population morph composition is not present throughout the geographic range of *U. ornatus* (Supplementary Fig. S4; see also Supplementary Fig. S3 for an emphasis on discrete morphs). The three color components are also expressed in most localities in my spatial dataset and there was a lack of spatial contiguity between the mono- or di-morphic localities. These observations suggest a limited role for population genetic structuring or dispersal limitation for driving observed morph diversity and distribution patterns. Interestingly, there appears to be spatial population genetic clustering among *U. ornatus* populations, with little to no dispersal between distinct clusters, as evidenced by their mtDNA^44^. If sexual selection is the primary driver of morph turnover^26^, then we would expect morph diversity patterns to also exhibit similar spatial clustering, which was not the case (Supplementary Figs. S3, S4). Alternatively, the concordance between my datasets supports a clear role for climate as a significant driver of turnover in all three color components, and thus the overall polymorphism, of *U. ornatus*. Data on the extent that genes associated with throat coloration are linked with any genes associated with environmental tolerance would be of great interest to this end.

Understanding the factors that may promote species persistence in the face of ongoing climate change is one of the most pressing challenges of our time. In color polymorphic species, population composition varies over time as morph frequencies fluctuate, and thus the gene pool available to face a given environmental challenge should also differ by year. Such temporal morph turnover may be adaptive if population turnover trends in favor of an optimal color phenotype over time: in my study, an example of this might be a warming locality where yellow-colored males become more common each successive generation. If competing morphs can also partition climatic space as my findings suggest, then polymorphic species should have a distinct advantage over monomorphic taxa in the face of rapid climate change^2^. And yet, at the same time, although the species itself may persist, the extent (or existence) of its color polymorphism should not because of those morph differences in the capacity to respond to similar climatic challenges. This process, although unexpected given predictions of polymorphism stability from a sexual selection perspective^26^, may already be underway at some localities for *U. ornatus*. Simply put, joint behavioral and environmental specialization may in some instances constrain, rather than promote, morph adaptive potential. These considerations challenge our understanding of the factors contributing to the maintenance and evolution of color polymorphisms, fostering a greater appreciation for the interplay between natural and sexual selection in polymorphic systems.

## Methods

### Study system

The ornate tree lizard (*U. ornatus*) ranges widely throughout western North America (Fig. 1b^45^) and thus its populations are exposed to a broad gradient of climate conditions that may influence morph turnover in addition to sexual selection. From a strictly phenotypic perspective, the color badge of *U. ornatus* and other ARS color polymorphic species is comprised of an outer and a central portion that may or may not match in color (Fig. 1a). Descriptions of the badge of color polymorphic species are thus based on observations of the color or combination of colors (typically denoted by uppercase letters) present in the badge^11,22,46,47^. From a genotypic perspective, evidence suggests that morph coloration is linked to a diploid combination of three putative color alleles (typically denoted by lowercase italicized letters) operating at a single locus^26^. Although originally described in the side-blotched lizard (*Uta stansburiana*)^26^, there is robust support for this model operating in *U. ornatus* as well. The throat color polymorphisms of *U. ornatus* and *U. stansburiana* are fixed at adulthood and both manifest as a pairwise combination of blue, orange, and yellow coloration (Fig. 1)^48^. Prior studies have shown that *U. ornatus* color morphs also have genetic basis and breed true^47,49^, and that multiple discrete color morphs (and their associated ARS) are ancestral to *Urosaurus*^50^. This long-term maintenance of morphs throughout their evolutionary history should not be expected unless selection for multiple morphs is as central to *Urosaurus* as it is in *Uta*^50^. In fact, an ancestral state reconstruction provides strong evidence that the *oby* allele system and associated polymorphism first described in *Uta*^26^ likely originated in the common ancestor to both *Urosaurus* and *Uta* lineages some 14–27 million years ago^48^. Thus, in *U. ornatus*, the six possible color morphs (B, O, Y, OB, YB, OY) likely represent diploid combinations of three putative color alleles (*b*, *o*, and *y*) (see Fig. 1a).

### Spatial dataset

During 2011–2017, I captured 601 adult (> 40 mm snout-vent length) male lizards by catch-pole during haphazard surveys across 58 localities distributed throughout the range of *U. ornatus* in the US (Fig. 1b, Supplementary Table S1). These localities span an extensive geographic gradient in terms of both latitude (~ 9° gradient, range: 29.3–38.3°) and elevation (~ 2177 m gradient, range: 37–2214.4 m) that includes the full range of macrohabitat types exploited by *U. ornatus* throughout its distribution in North America^45^. I identified the color morph of each male by visual inspection of their throat patch (Fig. 1a). Lizards were then marked with a paint spot at the base of their tail (to prevent recapture) and released at their respective capture points.

### Time-series dataset

My time-series data draw from three independent localities within the Appleton-Whittell Research Ranch (AWRR) in Santa Cruz County, Arizona (Supplementary Table S2). I captured 590 adult male *U. ornatus* by catch-pole at these localities over a seven-year period (2010–2016) during the breeding season of each year (ca. May–July) (Supplementary Table S2). As with my spatial dataset, I classified the color morph of each captured male by visual inspection of his throat patch. Lizards were then marked with a paint spot at the base of their tail (to prevent recapture) and released at their respective capture points.

### Climate change dataset

During June 2015, I visited the University of Arizona Museum of Natural History to collect historical data on the throat color of 139 adult male *U. ornatus* specimens drawing from 14 localities in southeastern Arizona (Supplementary Table S3). These localities were identified using detailed museum records; 13 of 14 localities overlapped with localities surveyed for the spatial dataset in 2015 (*n* = 116; Supplementary Table S3). All localities were similar in latitude but varied in elevation (latitude range: 31.39–32.42°; elevation range: 741–1732.1 m). Lizards were originally captured from these localities between 1952 and 1987 (Supplementary Table S3). Throat color identification was limited: of the three colors that may comprise a male’s throat badge, only blue remained identifiable in preserved specimens. Blue coloration involves structural pigments, unlike orange and yellow coloration which, in general, may be synthesized directly (pteridines) and/or derived from their diet (carotenoids). Based on observations of inter-annual consistency in individual O and Y throat coloration in a close relative, *U. graciosus*^6^, and studies of throat patch pigment composition in sister genera to *Urosaurus*^51,52^, O and Y coloration in *U. ornatus* is likely generated primarily by pteridine pigments which quickly fade during preservation. Because inferences related to long-term turnover of orange or yellow coloration were not possible, I scored each lizard specimen in this dataset based on the presence or absence of blue coloration on their throat.

### Climate variables

I obtained interpolated climate data (daily maximum air temperature [°C] and precipitation [mm]) at 1/8-degree resolution from the Downscaled CMIP3 and CMIP5 Climate and Hydrology Projections archive (https://gdo-dcp.ucllnl.org/downscaled_cmip_projections/^53^;) for each locality based on its geographic centroid in latitude and longitude. Downloaded climate data from this archive contain projections from several climate models for each variable; I averaged across all models to generate estimates of daily temperature and precipitation at each locality. These data were obtained for a one-year period prior to lizard capture (June of previous year through May of current year). From these data, I calculated the mean and standard deviation of daily maximum temperatures (°C), the absolute maximum temperature (°C), mean daily precipitation rate (mm/day), the coefficient of variation (sd/mean) of mean daily precipitation rate (%), and the total precipitation during the active season (March-September for *U. ornatus*, in mm; see^41^). I relied on this one-year period because it falls between the mean age at maturity and generation time for *U. ornatus* in Arizona^41^. Thus, these climate data should capture the conditions experienced by a given lizard (or morph) that contributed to its ability to survive to that point at that locality. To validate the use of interpolated climate data in my study, I assessed the correlation between the daily maximum temperature and precipitation recordings from 23 Arizona weather stations in the US Climate Reference Network of the National Oceanic and Atmospheric Administration (https://www.ncdc.noaa.gov/crn/) (Supplementary Table S4) with climate estimates interpolated at the latitude and longitude of each station for the 2013 study year (representing a temporal midpoint for the time-series dataset). Both projected temperature and precipitation variables were significantly correlated to their analogs derived from actual station recordings (both *r* > 0.8 and *p* < 0.001, see Supplementary Fig. S1).

For the time-series dataset, I obtained daily maximum air temperature data (°C, summarized from raw data recorded at 5-min intervals) directly from the Southwest Watershed Research Center’s weather station at the AWRR^54^. This station has been recording temperature (among other variables) at the AWRR since 1997 and is situated central to all three study localities (mean distance = 2.2 km; range = 1.3–3.9 km), and at similar elevation (range: 1457–1497 m). I obtained daily precipitation data from the same database as in my spatial dataset. Using these data, I generated the same climate variables over a one-year time frame up to each annual capture period in the same manner as described for my spatial dataset.

### Statistical analysis

I coded individual *U. ornatus* males based on the color composition of their throat badge (see Study system; see also Fig. 1a). My coding is consistent with both a phenotypic and genotypic approach to characterizing throat coloration in ARS color polymorphic lizards^26,55^. Both approaches converge on the treatment of the color badge of a male as a combination of two components that directly (phenotypic approach) or indirectly (genotypic approach) denote badge coloration. To recognize this overlap but avoid confusion between the terminology of these approaches, I hereafter focus on the combinations of blue, orange, or yellow color components that comprise a male’s color badge. This procedure entailed creation of three binary variables (one per color component) and, for each male, assigning a ‘1’ or a ‘0’ depending on the presence or absence of that component in its throat color, respectively. I did not treat color component frequency within each male because my interest was in the association between color component presence and climate characteristics (i.e., a B male would only receive a ‘1’ for the blue color component in my framework, even though its underlying genotype may be *bb*; see Fig. 1a). Dimorphic phenotypes were considered successes for both color components involved (e.g., a YB male would receive a ‘1’ for both blue and yellow color component variables). My approach is preferable to treating all six color morphs separately because it allowed me to (i) assess all instances where a color shows up, regardless of a male’s overall morph identity (i.e., both YB and OB males have blue coloration), (ii) consider the putative genetic and physiological architecture underlying morph color (i.e., prior studies have shown that any ‘blue’ enhances dominance status in *U. ornatus*^46,56^), and (iii) provide a conservative estimate of color-specific climate associations (if any).

#### Spatial dataset

Climate data were correlated to varying degrees (Supplementary Table S5), and thus I employed a Principal Components Analysis (PCA) to compress these data into two uncorrelated variables (hereafter SpatPC1 and SpatPC2). These two axes explained 83% of the variation in temperature and precipitation conditions across the studied area (Table 1). I relied on factor loading scores to determine whether any variable(s) explained variation in the PCA: only scores greater than the absolute value of the mean loading (0.402) were interpreted. SpatPC1 described variation in maximum temperature and precipitation, with higher values corresponding to hotter and drier conditions overall. In contrast, SpatPC2 represented a gradient of mean daily temperature and seasonality in temperature and precipitation, with higher values indicating environments experience constant, warmer temperatures but greater precipitation variability.

I tested for overall variation in morph frequency using a Chi-Square test. I evaluated the effects of climate on variation in morph color component occurrence using a series of generalized additive models (GAMs) with a binomial error distribution (function ‘gam’ in mgcv package in R^57^). Four candidate models were fit per color component: a saturated model including PC1 and PC2 as predictors, a PC1-only model, a PC2-only model, and a null model (intercept-only). I assessed model fit via likelihood-ratio tests (function ‘lrtest’ in package lmtest^58^;) and sample-size corrected AIC (AICc) values and weights (function ‘Weights’ in MuMIn package^59^). The model that significantly improved fit to the data relative to the null model and had the lowest AICc and the highest AICc weight was retained as the best-fit model^60^. For a given color component, models were tested against a two-column response matrix representing the number of successes (number of males exhibiting the color component) and failures (number of males lacking the color component) at each locality. In other words, these analyses focused on total color component frequency patterns at each locality (rather than individual organism color component composition), allowing me to assess patterns of color component turnover across localities (and thus populations). Assumptions for each candidate model were verified prior to interpretation (via the ‘gam.check’ function in the mgcv package^57^;). Finally, because my models were fit to binomial data, I described the explanatory power of the best-fit model for each color component using a sample-size adjusted Nagelkerke’s *R*^2^^61^, implemented via the function ‘r.squaredLR’ available in the MuMIn package^59^.

#### Time-series dataset

Climate variables from the ranch weather station were correlated to varying degrees (Supplementary Table S5); thus, I ran a PCA on these climate variables (Table 1). I retained the first two axes from the output, which explained 82.3% of the variance in climate, as climate variables in all subsequent analyses (hereafter TimePC1 and TimePC2). Only scores greater than the absolute value of the mean loading (0.404) were interpreted. TimePC1 described variation in mean and maximum temperatures, and mean precipitation, with higher values corresponding to hotter but wetter conditions. TimePC2 described variation in mean temperature and precipitation rates, and seasonality in those variables, with higher values indicating relatively constant hot, arid conditions.

I tested for overall variation in morph frequency via a Chi-Squared test. As with the spatial dataset, I used a series of binomial GAMs to evaluate the role of climate in temporal color component turnover (all functions, model designs, assumptions verification, and interpretation are qualitatively the same as described for the spatial dataset above).

#### Climate change dataset

For each locality-year combination (historical and present-day) for the 14 localities included in this dataset, I first determined the proportion of males with a blue color component in the sample (number of males with any amount of blue throat coloration divided by total number of males). I then calculated the shift in proportion of males with a blue color component over time as the proportion of blue males in the present-day sample minus the proportion of blue males in the historical sample. Here, values less than or greater than zero indicate a shift in population composition towards fewer or more males exhibiting blue coloration over time, respectively. I used the same approach to calculate shifts in each of the climate variables, and then I applied correlation tests to evaluate the extent that shifts in the population composition of blue males coincided with recent climate change.

### Ethical statement

All work was conducted in accordance with accepted guidelines for research using live reptiles (ASIH/HL/SSAR Guidelines for Use of Live Amphibians and Reptiles) and was approved by all relevant oversight agencies (IACUC: OU-R06-07, CNU-2015-18; Permits (ordered by year for AZ): AZ [SP795884, SP792912, SP790917, SP625078, SP673600, SP715013, SP750397, SP510761], NM [3498], TX [SPR-416-109], San Bernardino National Wildlife Refuge [22523-2011-03]).

## Supplementary Information

Supplementary Information 1.

Supplementary Information 2.

## Data Availability

Raw data for this study are available in the online supplementary material.
