# Supplementary material for: Climate mediates color morph turnover in a species exhibiting alternative reproductive strategies
Source: Sci Rep. 2022 May 19;12:8474. doi: 10.1038/s41598-022-12300-7 (PMC9120169; doi:10.1038/s41598-022-12300-7)

Supplemental Material for

Climate mediates color morph turnover in a species exhibiting alternative reproductive strategies

Matthew S. Lattanzio*
Department of Organismal and Environmental Biology, Christopher Newport University, Newport News, Virginia 23606
*Correspondence: Email: matthew.lattanzio@cnu.edu

Table S1.
Geospatial data and sample sizes (total number of male *U. ornatus*) for the 58 localities included in the spatial component of this study. Latitude and longitude are presented in decimal degrees. Locality codes provide a reference for Figure S3.

Locality	Locality code	Latitude	Longitude	Elevation (m)	Sample size
AguaCaliente	AC	32.28324	-110.7392691	820.22	17
AguaCalienteWest	AW	32.282383	-110.7402346	820.7	7
AguirreSprings	AG	32.369679	-106.5616463	1735.09	14
AppletonRanch	AR	31.613405	-110.5085064	1457.05	12
AppletonRanchHigh	AH	31.613362	-110.5088379	1457	22
AppletonRanchLow	AL	31.594374	-110.5248989	1493.9	22
AppletonRanchWest	AS	31.588507	-110.5089517	1458.4	6
Arivaca	AV	31.46749	-111.2672995	1291.74	10
BearCanyon	BC	31.39432	-110.3392288	1865.38	23
BearPicnic	BP	32.33706	-110.6915598	1317.35	4
BearTrail	BT	32.26374	-110.6467697	1182.01	6
BigBendRanchSP	BB	29.301362	-103.8462576	738.84	7
BoxCanyon	BX	31.8014	-110.7637696	1521.26	7
Calabasas	CL	31.39266	-111.0351896	1205.79	7
CampbellAve	CA	32.33725	-110.9276897	899.16	6
CampVerde	CV	34.5767669	-111.8520053	930.65	13
CanadaDelOro	CD	32.39466	-110.977019	756.82	10
CanadaDelOroB	CB	32.39466	-110.977019	756.82	9
CapitalReef	CR	38.2653043	-111.2573198	2056.48	4
Dodge	DO	32.27139	-110.9145498	725.5	4
FranklinMtn	FM	31.915143	-106.508282	1590.86	19
GardnerCanyon	GC	31.72107	-110.7170392	1497.79	10
GardnerCanyonB	GB	31.72107	-110.7170387	1497.79	10
GarnerSP	GS	29.595348	-99.7437995	547.57	9
GomezPeak	GP	32.843024	-108.2838284	2214.43	12

GuadalupeRiverSP	GR	29.867294	-98.4982335	344.43	9
HoughtonRd	HR	32.29067	-110.77257	822.66	6
LakeMary	LM	35.1927343	-111.5866854	2083.26	11
LoneStarMine	LS	31.83252	-110.3698795	1496.26	10
MalpaiRanch	MR	31.335467	-109.3017489	1161.7	22
McDonaldRanch	MD	31.47457564	-109.0908996	1438.3	26
MillerCanyon	MC	31.42647	-110.2550444	1554.2	17
MonumentValley	MV	37.0086359	-110.3067134	1642.16	11
NaturalBridges	NB	37.6024169	-110.0153182	1877.53	15
Nogales	NO	31.392896	-110.9850746	1225.96	8
NoonCreek	NC	32.66894	-109.7943428	1559.3	4
PantanoRiver	PA	32.2368	-110.8384896	774.9	8
PearceRd	PR	31.90709	-109.9043595	1435	9
PineryCanyon	PA	31.96973	-109.3219198	1732.1	3
PineryCanyonB	PB	31.97024	-109.3353298	1704.2	4
RillitoRiver	RR	32.26395	-110.8413795	754.38	13
RinconCreek	RC	32.13286	-110.7337098	877.21	9
Rodeo	RO	31.939532	-108.9503545	1357.43	4
RomeroCanyon	RM	32.42469	-110.9051595	860.15	9
SabinoCanyonRd	SR	32.31329	-110.8195497	835.15	9
SabinoDam	SD	32.3149	-110.8115293	822.96	6
SanBernardinoNWF	SB	31.3427	-109.265149	1139.8	13
SilverCity	SC	32.50918	-108.5388113	1753.83	19
Sunnyside	SU	31.43953	-110.4007297	1790.5	13
SycamoreCanyon	SY	31.3878	-110.6914697	1664.51	6
Tanque14	TA	32.26217	-110.6495448	1156.8	6
Tanque20	TN	32.325673	-110.5430438	1199.7	4
TanqueFalls	TF	32.25576	-110.6539194	956.46	4
TontoNF	TO	33.7696101	-110.9867415	1383.48	12
UniversityOfAZ	UA	32.23079	-110.9483436	743.9	10
Wickenburg	WI	33.90965	-112.6756304	586.85	8
Yuma	YU	32.728384	-114.6190824	37.02	12

Table S2.
Geospatial data, sample sizes (total number of male *U. ornatus*), and year sampled for the three localities at the Appleton-Whittell Research Ranch in Santa Cruz County, Arizona that are included in the time-series component of this study. Latitude and longitude are presented in decimal degrees.

Locality Code	Latitude	Longitude	Elevation (m)	Sample size	Year
HB	31.613948	-110.507013	1462	24	2010
				23	2011
				29	2012
				36	2013
				42	2014
				41	2015
				16	2016
LB	31.592579	-110.523047	1457	26	2010
				17	2011
				30	2012
				34	2013
				51	2014
				35	2015
				28	2016
NB	31.553948	-110.49631	1497	17	2010
				23	2011
				31	2012
				21	2013
				26	2014
				19	2015
				21	2016

Table S3.
Historical and present-day sample sizes of all male *U. ornatus* and lizards expressing blue coloration for the 14 localities included in the climate change dataset. All localities, except for UniversityOfAZ2, overlapped with localities included in my spatial dataset.

Locality	Historical total *n*	Present-day total *n*	Historical *n* blue males	Present-day *n* blue males
AguaCaliente	14	17	10	12
AguaCalienteWest	10	7	8	4
CampbellRd	4	6	2	5
SabinoCanyonRd	46	6	43	6
SabinoDam	15	13	13	12
UniversityOfAZ1	7	8	3	6
UniversityOfAZ2	6	8	3	6
RillitoRiver	6	9	3	9
RomeroCanyon	7	9	6	9
RinconCreek	5	4	4	3
PantanoRiver	5	8	3	4
PineryCanyon	5	6	4	6
SycamoreCanyon	5	6	5	6
PearceRd	4	9	3	6

Table S4.
Weather station information for the 23 Arizona stations included in my validation of the interpolated climate data used in my study (fig. S1). Variables include Climate Reference Network (CRN) ID, Station locality code, general locality, latitude and longitude (both in decimal degrees), and elevation.

CRN ID	Station locality code	Locality	Latitude	Longitude	Elevation (m)
53168	AJO_29_S	Organ Pipe Cactus National Monument	31.94	-112.8	506
53006	AMADO_23_W	Buenos Aires National Wildlife Refuge	31.69	-111.44	1006
3085	BOWIE_23_SS3	Chiricahua National Monument	32	-109.38	1565
53159	CAMERON_25_SSE	Wupatki National Monument	35.5	-111.34	1465
53160	CAMPVERDE_3_N	Montezuma Castle National Monument	34.61	-111.84	1047
53162	COOLIDGE_5_W	Casa Grande Ruins National Monument	32.99	-111.53	434
53132	ELGIN_5_S	Appleton-Whittell Research Ranch	31.59	-110.5	1466
53183	FREDONIA_7_SSE	Bureau of Land Management	36.85	-112.45	1569
53176	GILABEND_3_ENE	Gila Bend Municipal Airport	32.96	-112.66	238
53170	HEBER_3_SE	Sitgreaves National Forest	34.39	-110.56	2019
3077	HOLBROOK_17_ESE	Petrified Forest	34.82	-109.89	1711

		National Park			
53169	KAYENTA_16_WSW	Navajo National Monument	36.68	-110.54	2217
53180	KINGMAN_8_NE	Kingman Airport	35.25	-113.93	1044
53172	LAKEHAVASUCITY_19_SE	Buckskin Mountain National Park	34.25	-114.13	127
53184	MEADVIEW_7_N	Lake Mead National Recreation Area	36.09	-114.04	896
53164	PAGE_9_WSW	Glen Canyon National Recreation Area	36.86	-111.6	992
53156	PHOENIX_7_S	South Mountain Regional Park	33.34	-112.08	428
53181	SAFFORD_5_NNE	Safford Regional Airport	32.85	-109.63	968
53011	TSAILE_1_SSW	Dine College	36.29	-109.21	2155
53131	TUCSON_11_W	Arizona-Sonora Desert Museum	32.23	-111.16	833
53019	WHITERIVER_A_1_SW	Fort Apache Tribe	33.82	-109.98	1577
53155	WILLIAMS_35_NNW	Babbitt Ranches	35.75	-112.33	1826
53154	YUMA_27_ENE	Yuma Proving Ground	32.83	-114.18	189

Table S5.
Correlations (below-diagonal), variances (diagonal), and covariances (above-diagonal) among the climate variables used for the spatial and time-series datasets. Significant correlations at α = 0.05 are bolded. Sources of climate variables are described in Methods.

Variable	T_{Mean}	T_{SD}	T_{Max}	P_{Mean}	P_{CV}	P_{Active}
Spatial dataset						
T_{Mean}	9.755	-0.243	8.227	-0.523	0.13	-117.9
T_{SD}	-0.123	0.398	0.338	-0.146	-0.024	-38.364
T_{Max}	0.934	0.19	7.954	-0.691	0.087	-167.154
P_{Mean}	-0.426	-0.587	-0.623	0.155	-0.017	35.508
P_{CV}	0.354	-0.322	0.262	-0.368	0.014	-2.89
P_{Active}	-0.407	-0.656	-0.639	0.974	-0.265	8591.236
Time-series dataset						
T_{Mean}	0.294	0.026	0.4	0.004	-0.012	-2.401
T_{SD}	0.139	0.124	0.154	0.009	0.002	3.352
T_{Max}	0.719	0.427	1.056	0.013	-0.015	0.81
P_{Mean}	0.153	0.555	0.272	0.013	-0.001	0.461
P_{CV}	-0.78	0.174	-0.522	-0.414	-0.001	0.088
P_{Active}	-0.34	0.733	0.061	0.742	0.246	169.15

Table S6.
Results of generalized linear mixed effects models evaluating the effect of climate (SpatPC1 and SpatPC2) on the occurrence of blue, orange, and yellow color components by male morphs at only those localities with ≥10 lizards in the spatial dataset (n = 28). Nagelkerke's R^2 values are provided for the best-fit model for each allele (i.e., lowest AICc and highest w_i(AICc)), and likelihood ratio tests compare fit of a given candidate model to the null model. See Methods for variable descriptions. Although there appears to be a difference in outcome is for the yellow color component, the full model nonetheless had the same support as the best-fit model (ΔAICc < 2). Thus, the wholesale removal of low sample size localities does not qualitatively impact the conclusions drawn from analyses presented in the main paper (see Results, Table 2).

Model	Syntax	AICc	ΔAICc	w_i(AICc)	$R^2_{adj.}$	χ^2	P
Blue color component							
BL12	~s(PC1) + s(PC2)	130.9	0	0.961	0.919	69.87	<0.001
BL2	~s(PC2)	137.3	6.42	0.039		55.49	<0.001
BL1	~s(PC1)	181.8	50.94	<0.001		12.41	0.006
BL0	~1	187.1	56.25	<0.001		-	-
Orange color component							
OR12	~s(PC1) + s(PC2)	153.6	0	0.995	0.74	37.57	<0.001
OR1	~s(PC1)	165.1	11.5	0.003		20.23	<0.001
OR2	~s(PC2)	166.4	12.81	0.002		23.81	<0.001
OR0	~1	183.2	29.65	<0.001		-	-
Yellow color component							
YE2	~s(PC2)	143.5	0	0.514	0.593	25.02	<0.001
YE12	~s(PC1) + s(PC2)	143.6	0.1	0.479		33.5	<0.001
YE1	~s(PC1)	152	8.55	0.007		16.42	<0.001
YE0	~1	161.1	17.65	<0.001		-	-

Figure S1.
Correlations between actual daily maximum temperature (°C) and precipitation (mm) data recorded at 23 weather stations in the US Climate Reference Network of the National Oceanic and Atmospheric Administration and their analogs drawn from the Downscaled CMIP3 and CMIP5 Climate and Hydrology Projections archive. All data were downloaded for the 2013 study year (June 2012 – May 2013) and, for the purposes of this assessment, averaged over the year for each station-dataset combination (resulting in 23 data points per graph). Trendlines are provided for visualization purposes; correlation (Pearson's *r*) and *p*-values are also provided.

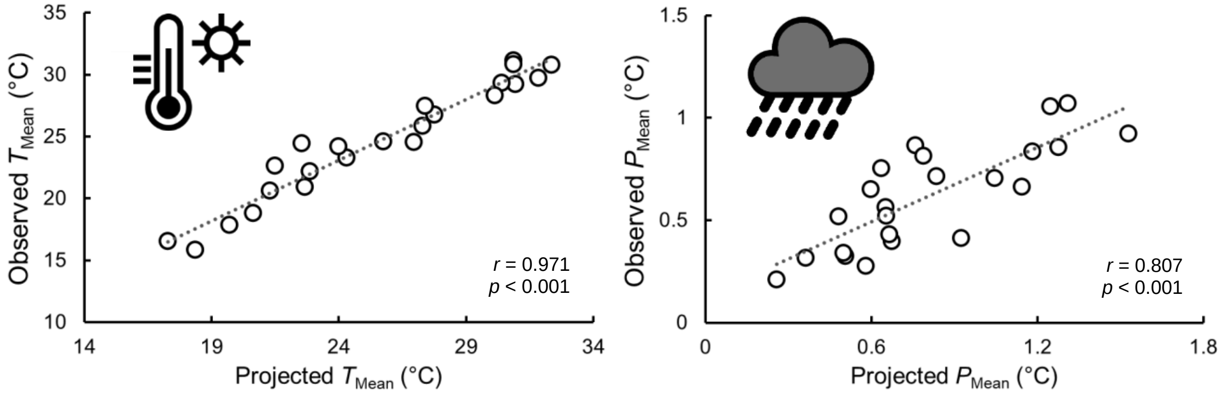

Figure S2.
Variation in microhabitat use by the 601 male *U. ornatus* included in my spatial dataset, focusing on the type of perch used by each lizard upon first encounter (trees, snags [=dead trees], or rocks). Here, a) provides data by morph and b) provides data by color component (blue, orange, or yellow). See Figure 1a for representative morph images. Overall, males with blue coloration preferred trees over other microhabitat types compared to other males (Chi-Square tests, by morph: $\chi^2 = 36.1$, df = 10, $P < 0.001$; blue color component: $\chi^2 = 10.4$, df = 2, $P = 0.006$; orange color component: $\chi^2 = 2.5$, df = 2, $P = 0.281$; yellow color component: $\chi^2 = 1.6$, df = 2, $P = 0.442$).

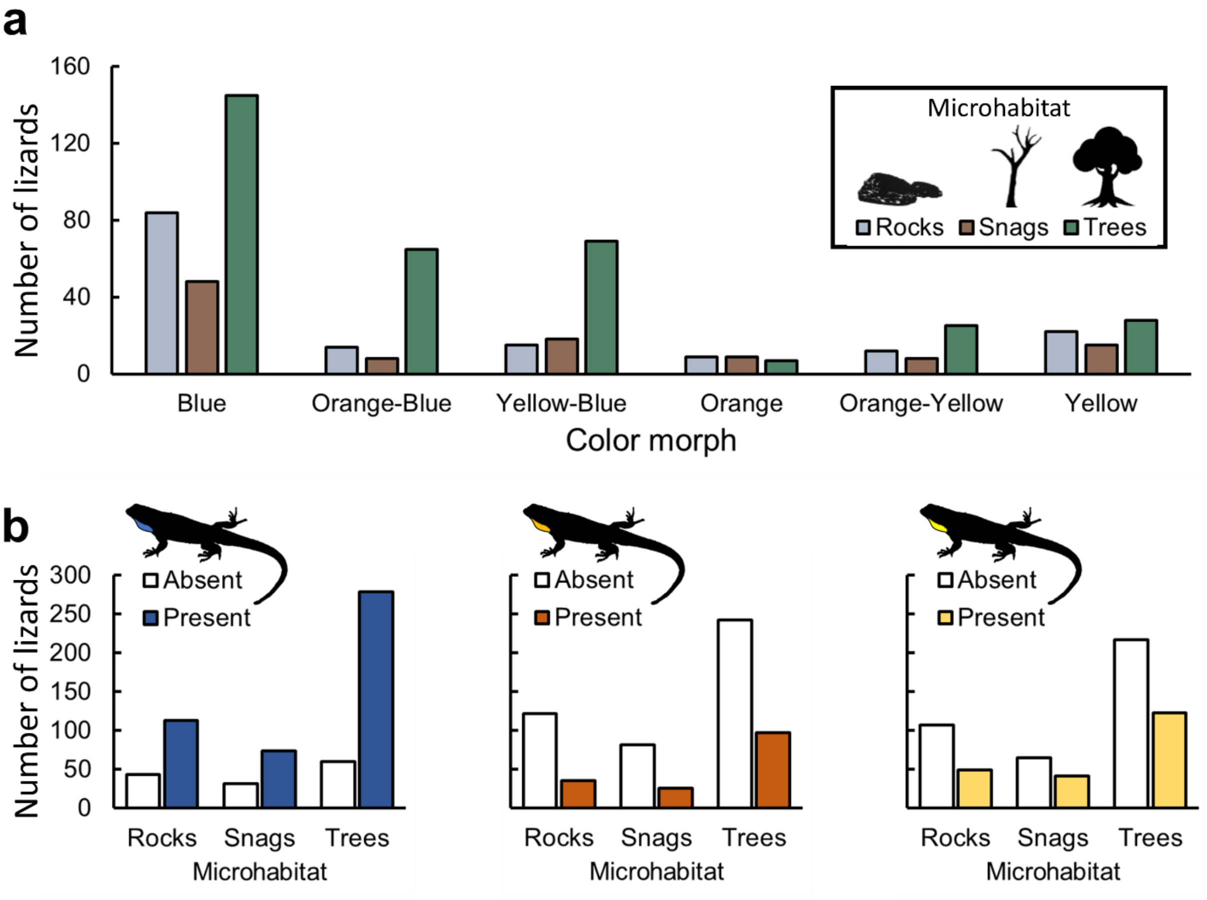

Figure S3.
Composition of male *U. ornatus* color morphs at each locality included in the spatial dataset. Localities are qualitatively ordered (i.e., not to scale) by longitude. Stacked localities share a similar longitude (~± 0.2°) and, for stacks only, locality height is loosely based on latitude (but again, not to scale). Refer to the legend inset to identify the color associated with each of the six color morphs (for morph photographs, see Figure 1), Figure 2 for a map of localities, and Table S1 for locality and sample size details. Overall, although there is a clear concentration of blue males in the eastern portion of the species' range, there is no consistent environmental cline associated with morph occupancy throughout the species' range (i.e., populations do not grade from all yellow or orange to all blue moving eastward; see also Figure S3 for lack of concordance between elevation and latitude gradients). Locality codes are as follows (ordered by longitude to match the figure below): YU = Yuma, WI = Wickenburg, CV = CampVerde, LM = Lake Mary, AV = Arivaca, CR = CapitalReef, CL = Calabasas, TO = TontoNF, NO = Nogales, CD = CanadaDelOro, CB = CanadaDelOroB, UA = UniversityOfAZ, CA = CampbellAve, DO = Dodge, RM = RomeroCanyon, RR = RillitoRiver, PA = PantanoRiver, SR = SabinoCanyonRd, SD = SabinoDam, HR = HoughtonRd, BX = BoxCanyon, AW = AguaCalienteWest, AC = AguaCaliente, RC = RinconCreek, GC = GardnerCanyon, GB = GardnerCanyonB, BP = BearPicnic, SY = SycamoreCanyon, TF = TanqueFalls, TA = Tanque14, BT = BearTrail, TN = Tanque20, AL = AppletonRanchLow, AS = AppletonRanchWest, AH = AppletonRanchHigh, AR = AppletonRanch, SU = Sunnyside, LS = LoneStarMine, BC = BearCanyon, MV = MonumentValley, MC = MillerCanyon, NB = NaturalBridges, PR = PearceRd, NC = NoonCreek, PO = Portal, PB = PineryCanyonB, PC = PineryCanyon, MR = MalpaiRanch, SB = SanBernardinoNWF, MD = McDonaldRanch, RO = Rodeo, SC = SilverCity, GP = GomezPeak, AG = AguirreSprings, FM = FranklinMtn, BB = BigBendRanchSP, GS = GarnerSP, GR = GuadalupeRiverSP.

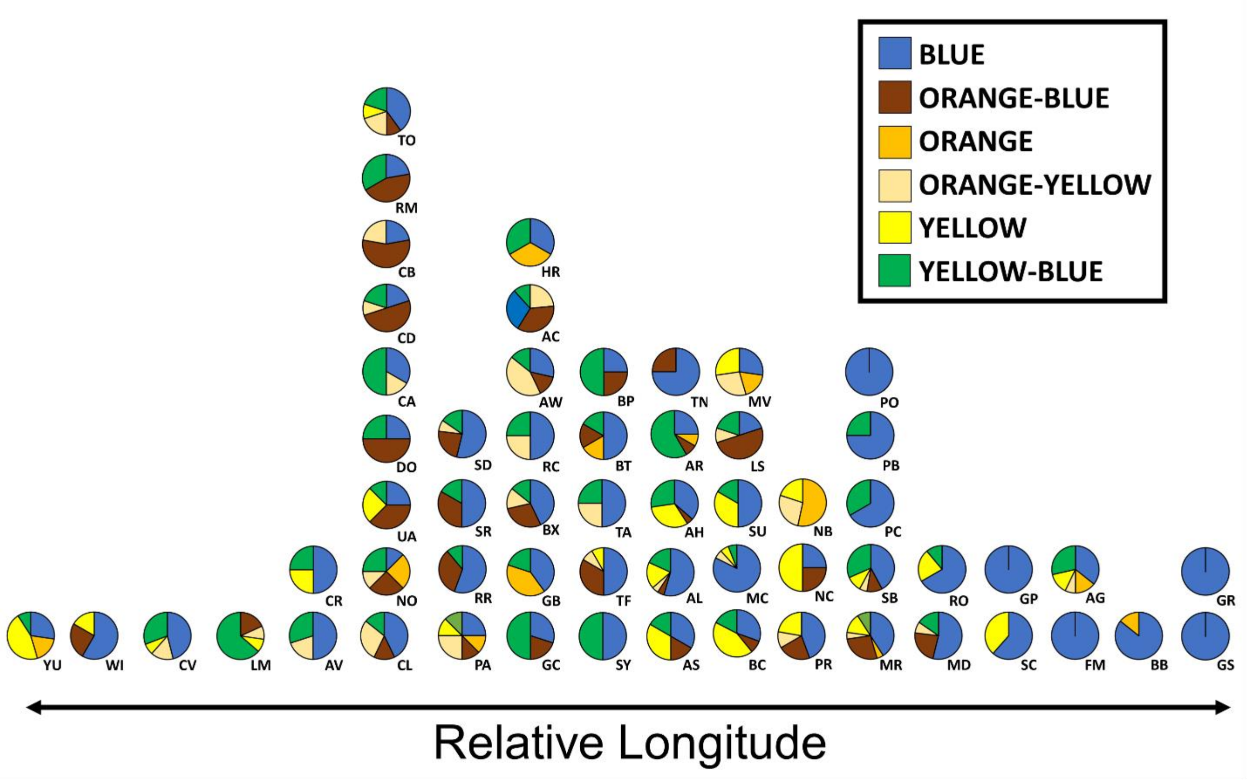

Figure S4.
Relationship of the proportion of male *U. ornatus* at each locality (*n* = 58) expressing a given color component (blue [b], orange [o], or yellow [y]) with latitude (in decimal degrees) and elevation (in meters), respectively. Most patterns were non-significant, or when significant, no consistent environmental clines across latitude and elevation were detected. Trendlines are for illustrative purposes only; *r* and *p* values are results of Spearman's rank correlation tests because most variables failed to meet assumptions of normality.

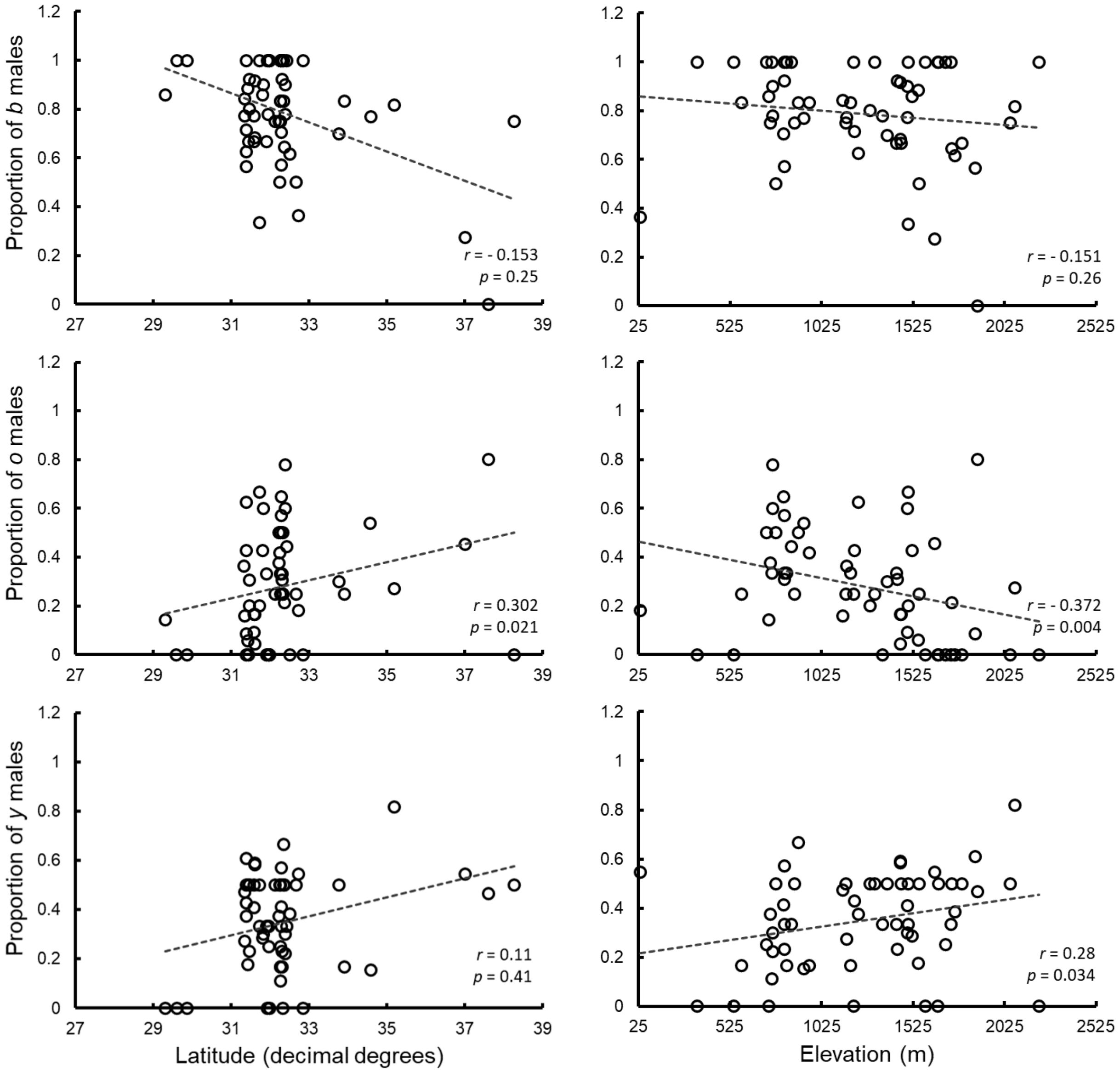

Supplement: Supplementary file 2 — Supplementary Information 2. [file 41598_2022_12300_MOESM2_ESM.pdf]
